# A comparison of operating room toric placement tools: CALLISTO eye vs. e Wavetec AnalyzOR (CORTCO)

**DOI:** 10.1186/s12886-024-03723-z

**Published:** 2024-10-28

**Authors:** Maria C. Scott

**Affiliations:** Chesapeake Eye Care and Laser Center, LLC Sajak Pavilion, 2002 Medical Parkway Ste. 320, Annapolis, MD 21401 USA

**Keywords:** Astigmatism, Callisto eye, WaveTec ORA, toric intraocular lens

## Abstract

**Background:**

To evaluate procedure times for two cataract planning systems (ZEISS CALLISTO eye and the Wavetec AnalyzOR) in predicting residual astigmatism (prediction error) and other visual outcomes in patients with corneal astigmatism (maximum allowable up to 3.0D) at postoperative month 1.

**Methods:**

This was a prospective, single center, parallel treatment group, bilateral and unilateral, randomized, 1-month study on patients scheduled to undergo routine, small-incision cataract surgery with a toric intraocular lens implantation. Both groups underwent preop measurements with the IOLMaster 700 (Zeiss, Jena, Germany) and surgery with the LenSx device (Alcon). Lens selection in the CALLISTO eye group was based on Zeiss VERACITY Surgery Planner (a web-based tool) and on the Wavetec AnalyzOR component of the ORA system (a real-time intraoperative aberrometer) for those eyes in the ORA group. All procedure and intraoperative times were measured with a stopwatch. Postoperative visual outcomes were evaluated between 1 and 2 months after surgery.

**Results:**

There were 23 eyes in the CALLISTO group and 28 eyes in the ORA group. The mean surgical time for the CALLISTO group was 28.09 ± 1.72 min compared to 34.41 ± 1.52 min for the ORA group (*P* = 0.01). Toric lens placement mean time in the CALLISTO group was 2.47 ± 0.34 min compared to 3.88 ± 0.29 min in the ORA group (*P* = 0.0034). At month 1 postoperatively, the manifest refractive spherical error (MRSE) in the CALLISTO eye group 0.022 ± 0.388 diopters (D) compared to -0.174 ± 0.322 D in the ORA group; these were not statistically different. There was a higher percentage (75%) of eyes with an MRSE within 0.25D in the ORA group compared to the CALLISTO eye group (56.5%); at all other levels outcomes were numerically higher in the CALLISTO eye group.

**Conclusions:**

Less surgical time was needed when using the CALLISTO eye than the ORA when performing cataract surgery with toric lens implantation. There were similar visual outcomes between the groups and no statistical differences.

## Introduction

Toric intraocular lenses (IOLs) provide a lens-based solution to correct astigmatism at the time of cataract surgery. Even patients with lower levels of astigmatism may benefit from these advanced technologies, with published studies and meta-analyses suggesting astigmatism from as low as 1 D to higher ranges of 6 D to 9 D can be effectively treated with a lens-based option [1–7]. Yet surgeons in the U.S. have not fully embraced toric lenses, partially due to the inability to precisely plan treatments and/or align the lens during surgery (as it is well accepted that reducing postoperative cylinder relies on a well-oriented toric lens) [8]. To aid surgeons in planning a toric IOL implantation is another technology advance: the introduction of image-guided surgery and intraoperative biometry (in addition to advanced IOL calculation formulas). Studies have shown using image-guided surgery streamlines the refractive cataract procedure [9] while producing more accurate spherical equivalent outcomes in eyes implanted with toric lenses to correct low levels of astigmatism [10]. However, Hovanesian advised caution when interpreting intraoperative readings if those readings disagreed by more than 0.5 D from preoperative biometry measurements [11]. 

The CALLISTO Eye (Carl Zeiss Meditec, Jena, Germany) provides markerless axis alignment to provide surgeons with a more precise and efficient toric IOL alignment, potentially reducing residual astigmatism [12–14]. The Wavetec AnalyzOR component of the Optiwave Refractive Analysis (ORA; Alcon) uses intraoperative measurements to allow surgeons to adjust both lens selection and the amount of rotation [15]. 

To date, real-world comparisons of surgical time between the CALLISTO Eye and the ORA when using them for toric lens implantation during cataract surgery have not been adequately explored. There remains a need to determine both the time it takes to perform the surgery and whether the additional time needed to use the ORA intraoperatively results in better visual outcomes postoperatively. Some have argued that toric IOL implantation surgeries may benefit the most from intraoperative aberrometry [16], but others have suggested little clinical benefit from the use of the technology [16–18]. 

The purpose of this study was to evaluate the amount of procedure time needed with each of these cataract planning tools for phakic power calculation and toric positioning to predict the residual astigmatism (prediction error) and other visual outcomes in patients with corneal astigmatism (up to 3.0D).

## Methods

### Study design

This was a prospective, single center, parallel treatment group, bilateral and unilateral, randomized, 1-month study on subjects who were eligible for a toric lens implant at the time of their planned cataract surgery. This study was conducted in accordance with U.S. Code of Federal Regulations, the Declaration of Helsinki, ISO 14155:2011 and all other applicable laws and regulations. The study was approved by Salus Institutional Review Board (Austin, Texas) and was retrospectively registered on ClinicalTrials.gov NCT06216067 (registration date: December 20th, 2023). All subjects had planned routine, small-incision cataract surgery and were eligible for implantation with the Tecnis toric II (Johnson & Johnson Vision, Santa Ana, Calif.). Up to four study visits were scheduled: preoperatively for one or both eyes examined together, operative visit for each eye (if being bilaterally implanted), and postoperative month 1 visit for each eye.

### Inclusion/exclusion criteria

Among the inclusion criteria: clear intraocular media, other than cataract; unilateral or bilateral cataract extraction with a single piece hydrophobic acrylic posterior capsular IOL to correct astigmatism, and an ability to complete all required visits and comprehend/sign an informed consent statement. Key exclusion criteria: ocular disease or pathology that would affect postoperative visual acuity (VA) and manifest refraction; any prior intraocular or corneal refractive surgery, corneal transplant, or retinal detachment.

### Subject randomization

Subjects were randomized by stratifying preoperative cylinder ranges corresponding to the lens being implanted. If the subject was eligible for bilateral implantation, the second eye was assigned to the same operating room as the first eye; if the subject was eligible for unilateral implantation only, randomization was adjusted so that the percentage of patients undergoing surgery with the CALLISTO eye was within 5% of those undergoing surgery with the Wavetec ORA.

### Surgical and examination procedures

Within 120 days prior to first eye surgery, preoperative data to determine eligibility was collected and informed consent was completed by all subjects. All preoperative outcomes were measured with the IOLMaster 700 (Carl Zeiss Meditec). Lens selection for the patients randomized to the CALLISTO was based on the Zeiss VERACITY Surgery Planner (a web-based tool) and on the Wavetec AnalyzOR component of the ORA system (a real-time intraoperative aberrometer) for those eyes in the ORA group. The surgeon performed standard, small-incision cataract surgery and only used validated implantation systems for lens implantation. Refractive target outcomes were emmetropia (closest to plano spherical equivalent) for all eyes. All eyes were implanted with the TECNIS^**®**^ Toric II IOL and all surgeries were performed using the LenSx device (Alcon, Ft. Worth, Texas). The second eye, if applicable, was implanted within 45 days of the first-eye surgery.

### Study endpoints

The primary endpoint was the amount of time each cataract planning system takes, including preoperative and intraoperative calculations; these were ascertained via a stopwatch. The primary efficacy endpoint was the percentage of patients who were within ± 0.5D of residual cylinder at postoperative month 1. Other outcomes included cylinder, uncorrected and best-corrected distance visual acuities (UDVA and CDVA) and rate of adverse events. All postoperative refractive outcomes were measured using the IOLMaster 700.

### Statistical analysis

All statistical analyses were performed with SAS Software, version 9.4 (SAS Institute, Cary, NC). It was anticipated that 50 eyes would provide > 90% power, defined using an equivalence margin of 0.5D of cylinder, to determine equivalence.

Baseline data was summarized for each group and compared statistically to determine comparability of the two treatment groups. Clinical outcomes were assessed between one- and two-months post procedure. The primary efficacy endpoint was residual astigmatism, measured in diopters. It is hypothesized that both ORA and CALLISTO were equivalent, using a margin of 0.5D.

For the analysis of continuous predicted residual astigmatism, a mixed-effects model analysis of variance model was used to address the dependence arising between eyes from subjects with bilateral treatment. For binary (responder) endpoints, dependence was handled with the use of general estimating equation models.

## Results

### Subject demographics

There was a total of 36 patients and 51 eyes enrolled, with slightly more eyes assigned to the ORA group (*n* = 29) than the CALLISTO eye (*n* = 21). See Table 1 for baseline patient characteristics.

**Table 1:** Baseline patient characteristics

	ORA (*N* = 29)	CALLISTO (*N* = 21)
No. of patients	19	17
Mean age (in years)	67.94	74.15
No. of males	7	4
No. of females	12	13
Type of astigmatism (no. of patients)		
With-the rule	2	4
Against-the-rule	16	10
Oblique	1	3

### Surgical time

**Fig. 1:**
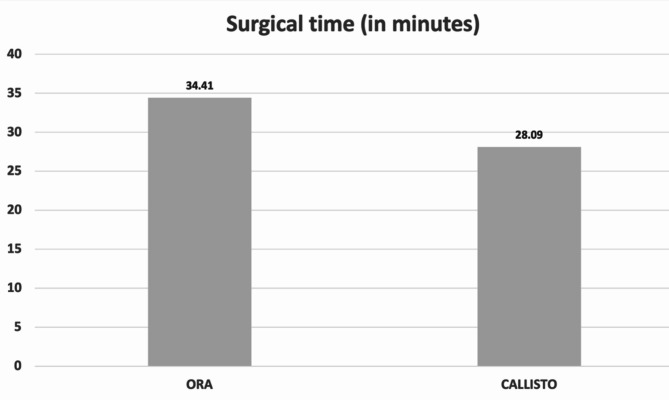
Surgical time differences between CALLISTO and ORA

The surgical time in the CALLISTO group ranged from 24.55 to 31.63 min, while the mean surgical time in the ORA group ranged from 31.29 to 37.53 min. The mean surgical time for the CALLISTO was 28.09 ± 1.72 and the mean surgical time for the ORA was 34.41 ± 1.52, with a mean difference of 6.23 min (*P* = 0.010; least squares mean [LSMeans]); see Fig. 1. Toric IOL placement was slower by 1.4 min when using the ORA compared to the CALLISTO (3.88 ± 0.29 min vs. 2.47 ± 0.34 min; *P* = 0.003). Other components of the surgery (suction time and total phacoemulsification time) showed no difference, statistical or numerical, as might be expected as all patients underwent surgery with the same phacoemulsification machine.

### Refractive outcomes

There was no statistical difference in uncorrected or best corrected distance visual acuity, and all eyes were 20/25 or better. At the postoperative month 1 visit, the manifest refractive spherical equivalent (MRSE) for eyes in the CALLISTO group was 0.022 ± 0.388 D and − 0.174 ± 0.322 D for eyes in the ORA group. All eyes were within 1 D. There were more eyes in the CALLISTO group (*n* = 23; 100%) that were within 0.75 D compared to eyes in the ORA group (*n* = 27; 96.4%) and more eyes in the CALLISTO group (*n* = 21; 91.3%) that were within 0.5 D compared to eyes in the ORA group (*n* = 24; 85.7%). However, there were fewer eyes in the CALLISTO group (*n* = 13; 56.5%) that were within 0.25 D compared to the number of eyes in the ORA group (*n* = 21; 75%). None of these differences were statistically significant.

Also at the month 1 follow-up visit, all eyes were within 1.0 D of target cylinder. The mean cylinder for eyes in the CALLISTO group (*n* = 19) was − 0.263 ± 0.452 D compared to a mean cylinder of -0.464 ± 0.576 D in the ORA group (*n* = 28). A higher percentage of eyes in the CALLISTO group (*n* = 14; 73.7%) were within 0.25 D of target compared to eyes in the ORA group (*n* = 13; 46.42%) and within 0.5 D of target [CALLISTO group (*n* = 14; 73.7%); ORA group (*n* = 13; 46.42%)]. These differences were not statistically significant.

### Visual outcomes

At 1-month post-surgery, UDVA was 0.018 ± 0.070 logMAR in CALLISTO group (*n* = 23) and 0.025 ± 0.071 in ORA group (*n* = 28) (Table 2). Moreover, 91.3% of the patients had a UDVA of 20/20 or better in CALLISTO group, and 96.4% in ORA group. All patients had a CDVA of 20/25 or better in both groups. No statistical differences in terms of UDVA or CDVA were found between each group.

**Table 2:** Postoperative visual acuities

	ORA (*N* = 23)	CALLISTO (*N* = 28)	*p* values
UDVA at 1 month, LogMAR			
Mean ± SD	0.025 ± 0.071	0.018 ± 0.070	0.9520
Min; Max	-0.120; 0.260	-0.120; 0.160	-
**CDVA at 1 month**,** LogMAR**			
Mean ± SD	0.006 ± 0.028	-0.002 ± 0.049	0.7480
Min; Max	-0.080; 0.100	-0.120; 0.100	-

CDVA, best-corrected distance visual acuity; SD, standard deviation; UDVA, uncorrected distance visual acuity.

### Safety outcomes

There were no adverse events reported

## Discussion

This was a real-world comparison of the amount of surgical time needed with either the CALLISTO eye or the ORA to perform cataract surgery with a toric IOL implantation, and whether or not there were any differences in postoperative outcomes between the two groups. In our practice, we often combine the use of CALLISTO eye with the ORA when planning for a toric lens implantation. Anecdotal evidence acknowledges extra time needed when implementing ORA [19, 20], but clinical studies suggest large discrepancies between preoperative biometry and intraoperative evaluations may be cause for concern [11], and may add even more time to the surgery. In this study, surgery with the ORA took about 6 min longer, but visual outcomes were similar between the two groups, or slightly favored the CALLISTO group.

Previous studies comparing image-guided surgery to traditional surgical procedures suggest the former can streamline refractive cataract surgery [9] and provide better outcomes in eyes with low level toric IOLs [10]. Anecdotal evidence acknowledges the extra time needed when using ORA, [[https://www.clevelandeyeclinic.com/cataracts-ohio/ora-system/]] [[https://www.carolinaeyemd.com/wp-content/uploads/2017/03/ORA-FAQs.pdf]] and our study adds to the growing data.

This study also adds to the literature on comparing ORA and CALLISTO: Solomon et al. compared residual refractive astigmatism and mean absolute error after cataract surgery and toric IOL implantation when the CALLISTO eye was used (*n* = 24) or ORA was used (*n* = 36) [8]. In that study, 97% of the eyes were 20/20 in the CALLISTO group compared to only 83% of eyes in the ORA group. Comparable to our study, Solomon et al. found when evaluating postoperative residual refractive cylinder the CALLISTO eye performed better at 0.25 D (71% vs. 36%)^8^; in our study, 73.7% and 46.2% were within 0.25 D of cylinder for the CALLISTO eye and ORA, respectively.

The CALLISTO eye has been evaluated at higher levels of preoperative astigmatism as well (albeit without toric lens implantation). Cao et al. [21] evaluated 76 eyes with corneal astigmatism > 0.75D using CALLISTO eye for the arcuate keratotomy and found the uncorrected distance VA logMAR was substantially better in the group that was assigned to the CALLISTO (0.15 ± 0.12) compared to the control group (0.24 ± 0.17); *P* < 0.01.

This study is not without limitations, among them the small sample size limiting the generalizability of the results, and absence of a control group. The advantage of randomization and the use of a single surgeon helped reduce bias and variability, enhancing the reliability of the findings in this study. However, the use of a single surgeon also represents a limitation in this study, since the reliability between multiple surgeons cannot be assessed. We also limited this study to include only patients with < 3.0 D of astigmatism, and therefore cannot extrapolate these findings to any group with > 3.0 D of astigmatism. Finally, only visual outcomes were assessed in this study, other outcomes such as ocular surface damage, inflammation, or retinal damage were not assessed in this study.

Busy refractive cataract practices are constantly looking for better efficiencies without compromising patient care. However, if improved patient care comes at the cost of more chair time or more surgical time, the trade-off may be worthwhile. This small study may help surgeons determine whether or not the use of the ORA is advantageous when planning for a toric lens implantation. In our real-world analysis, surgical times were longer with the ORA (by about 6 min) than with the CALLISTO, and visual outcomes were slightly better for eyes that had been treated using the CALLISTO eye than with intraoperative aberrometry. However, multicenter studies involving multiple surgeons and a larger population size need to be performed to confirm these results and to assess the inter-surgeon variability.

## Conclusion

Using the CALLISTO eye resulted in a quicker surgical time (6.23 min faster) than using the ORA, with similar visual outcomes and no statistically significant differences.

## Data Availability

The datasets used and/or analysed during the current study are available from the corresponding author on reasonable request.

## Abbreviations

IOL: Intraocular lens
VA: Visual acuity
